# Mr. Nixon's Resignation at the London Hospital

**Published:** 1892-09-24

**Authors:** 


### HOSPITAL WORTHIES.

### MR. NIXON'S RESIGNATION AT THE LONDON HOSPITAL.

A Special General Court of the Governors of the London Hospital was held on Tuesday, the 20th inst., to formally receive Mr. Nixon's resignation of the house governorship, and to elect a successor. Mr. E. Murray Ind, chairman of the House Committee, presided.

On the motion of the Chairman, the resolutions passed at the Special Court on the 7th inst. altering the bye-laws so as to allow of the amalgamation of the offices of House Governor and Secretary was confirmed.

The Chairman having read the letter given last week from Mr. Nixon resigning his office, said that he now had formally to move that Mr. Nixon's resignation be accepted, and that he be granted a retiring pension of £750 a year. To what had been said at the previous Court he could only add that the loss to the London Hospital would be a truly great one. In Mr. Nixon they were losing a man whom it would be very hard to replace, a man who had seen the hospital grow from a small institution to a large one, and a man who, great as the growth had been, had proved himself well able to carry on the increased duties.

The resolution was seconded and carried unanimously.

The Chairman said the next resolution he had to move needed no comment. It was as follows :—

"That the Special Court of Governors held at the London Hospital on the 20th inst. to receive the resignation of Mr. W. J. Nixon unanimously decide to place on record their thorough appreciation of the immense success with which he has carried out the arduous and delicate duties of House Governor since 1866, and of the great benefit that has accrued to this charity from Mr. Nixon's intimate connection with it since he was appointed Secretary in July, 1846. The devotion and courtesy with which Mr. Nixon has carried on the work of the London Hospital, and the deep sympathy with which he has interested himself in the sufferings and needs of the poor have gained for him the deepest gratitude of the Governors of this hospital. In conclusion, the Governors earnestly wish Mr. Nixon a speedy and complete restoration to health and long life to enjoy his well-earned rest."

Mr. Carr-Gomm in seconding, said that he had, as Chairman of the hospital for some five years, been brought into very close contact with Mr. Nixon. Any success he might have had was due in a great measure to the assistance derived from the wonderful knowledge of all hospital matters, and the experience which Mr. Nixon possessed. As an official under the Government, he (Mr. Carr-Gomm) had in his time been brought into contact with many people and many officials, but never with one who had had a more intimate acquaintance with every detail of a large institution than Mr. Nixon. All present no doubt took a great interest in the London Hospital, but in Mr. Nixon's case the hospital was his life's work—he had lived for the hospital entirely. Not only did Mr. Nixon do an immense amount inside the hospital, but it was a great deal due to the confidence that the public were able to repose in him and his administration that the hospital had received so very large an amount of support. He expressed a hope that Mr. Nixon would live to enjoy his pension for many years.

Mr. Hall, in supporting, spoke from experience of the high estimation in which Mr. Nixon was held in the East of London.

Mr. Spencer Charrington, M.P., said he had known Mr. Nixon nearly ever since he took office as secretary. He recollected, when it was suggested that the office of Secretary and House Governor should be combined on the retirement of Mr. Hill, asking Mr. Hill whether any one man could do the work. Mr. Hill replied, "Nixon can do it, but I do not think any other man can." Mr. Nixon, as they knew, did do it. A more efficient head of a hospital than Mr. Nixon never existed, and they all very deeply regretted to find that his retirement was caused not only through old age but by ill-health. They hoped that he would have many more years of life credited to him.

A letter from Mr. Henry C. Burdett was read by the Chairman. After regretting that absence from London prevented him from being present, Mr. Burdett stated he had been associated with the administration of hospitals for nearly thirty years, and during that period he had known Mr. Nixon's work, and had had cause to appreciate its special value. No man had rendered more faithful service than Mr. Nixon. Always courteous, obliging, and absorbed in the work of the London Hospital, he would ever remain a prominent figure among the worthies who had devoted their lives to the cause of the suffering poor.

The resolution was carried unanimously.

The Chairman having declared the office of House Governor vacant, said that when the House Committee arrived at the decision of coupling the two offices of Secretary and House Governor, they felt that they were really putting a great deal of work and responsibility on to one man. They had the advantage, however, of knowing that since Mr. Nixon had been laid up, *i.e.*, since April, Mr. Roberts had had all the responsibilities, if not all the duties, of the two offices, and they had had cause to believe that it was to the benefit of the institution to have one man at the head of affairs. The House Committee felt that they were making the best proposition that it was possible to make in proposing this amalgamation and appointment. Mr. Roberts, who it was proposed should fill the joint office, had been Secretary to the hospital for four and a half years, and the Committee, who had an intimate knowledge of his work, had full confidence in recommending his appointment. He therefore formally moved Mr. Roberts' election as House Governor.

Mr. Spencer Charrington, M.P., in seconding, said that the way in which Mr. Roberts had done his duties as Secretary had gained for him such a very high position in the opinion of the House Committee that he had been unanimously chosen for the post.

The resolution was carried unanimously.

Mr. G. Q. Roberts, in returning thanks, said that he quite realised the very great honour which had been done him. He recognised that very heavy responsibilities rested on him, but he hoped he would be able to carry out the work to the complete satisfaction of all.

The proceedings then terminated.

